# From Forest to Farm: Systematic Review of Cultivar Feeding by Chimpanzees – Management Implications for Wildlife in Anthropogenic Landscapes

**DOI:** 10.1371/journal.pone.0033391

**Published:** 2012-04-11

**Authors:** Kimberley J. Hockings, Matthew R. McLennan

**Affiliations:** 1 Departmento de Antropologia, Universidade Nova de Lisboa, Lisboa, Portugal; 2 Centre for Research in Anthropology (CRIA), Lisboa, Portugal; 3 Anthropology Centre for Conservation, Environment and Development, Oxford Brookes University, Oxford, United Kingdom; University of California, Berkeley, United States of America

## Abstract

Crop-raiding is a major source of conflict between people and wildlife globally, impacting local livelihoods and impeding conservation. Conflict mitigation strategies that target problematic wildlife behaviours such as crop-raiding are notoriously difficult to develop for large-bodied, cognitively complex species. Many crop-raiders are generalist feeders. In more ecologically specialised species crop-type selection is not random and evidence-based management requires a good understanding of species' ecology and crop feeding habits. Comprehensive species-wide studies of crop consumption by endangered wildlife are lacking but are important for managing human–wildlife conflict. We conducted a comprehensive literature search of crop feeding records by wild chimpanzees (*Pan troglodytes*), a ripe-fruit specialist. We assessed quantitatively patterns of crop selection in relation to species-specific feeding behaviour, agricultural exposure, and crop availability. Crop consumption by chimpanzees is widespread in tropical Africa. Chimpanzees were recorded to eat a considerable range of cultivars (51 plant parts from 36 species). Crop part selection reflected a species-typical preference for fruit. Crops widely distributed in chimpanzee range countries were eaten at more sites than sparsely distributed crops. We identified ‘high’ and ‘low’ conflict crops according to their attractiveness to chimpanzees, taking account of their importance as cash crops and/or staple foods to people. Most (86%) high conflict crops were fruits, compared to 13% of low conflict crops. Some widely farmed cash or staple crops were seldom or never eaten by chimpanzees. Information about which crops are most frequently consumed and which are ignored has enormous potential for aiding on-the-ground stakeholders (i.e. farmers, wildlife managers, and conservation and agricultural extension practitioners) develop sustainable wildlife management schemes for ecologically specialised and protected species in anthropogenic habitats. However, the economic and subsistence needs of local people, and the crop-raiding behaviour of sympatric wildlife, must be considered when assessing suitability of particular crops for conflict prevention and mitigation.

## Introduction

With the large-scale and accelerating conversion of natural habitats to alternative land-uses including farming, wildlife populations are increasingly exposed to cultivated foods [1], [2]. Globalisation means that new foods, especially cash crops, are being introduced into geographical areas where they were previously absent. Crops are palatable, energy-rich, easily digestible, and often clumped in spatially abundant fields or plantations. Consequently, crops offer energetic advantages over many natural foods for wildlife in agricultural–forest ecotones [3], [4]. Certain wildlife species can adapt their feeding ecology to exploit anthropogenic habitats, including cultivated landscapes, by incorporating cultivars into their diets, e.g. Elephant, *Loxodonta africana* [5]; Racoon, *Procyon lotor* [6]; Baboon, *Papio anubis* [7]; Hippopotamus, *Hippopotamus amphibius* [8]. Although some cultivars are obtained from abandoned or naturalised sources, crop-feeding by wildlife often involves an animal venturing into a cultivated area such as a field, plantation or orchard and exploiting foods that humans perceive as belonging to them.

Crop-raiding is a major source of conflict between wildlife and people globally [2]. Crop-raiding compromises biodiversity conservation initiatives by generating negative perceptions of wildlife and may threaten rural people's economic security [9]. Such negative perceptions, especially concerning large-bodied species such as elephants and great apes, can be exacerbated by the potential risk they pose to human safety [10]–[12]. As a result, crop-raiding animals risk harassment, injury or even death during confrontations with people. This includes taxa that are endangered and legally protected, including great apes (chimpanzees *Pan troglodytes* [13]), gorillas *Gorilla berengei* [14], orangutans *Pongo* spp. [15]).

The mitigation of human–wildlife conflict requires evidence-based management [16]. When anthropogenic impacts on animal behaviour cause conservation concerns, a behavioural-based management approach is recommended to inform appropriate management strategies (e.g. land-use changes, reserve design or corridor planning) based on the species' behaviour or to alter the behaviour directly [17]. In cognitively complex species, changing problematic behaviour such as crop-raiding can be extremely difficult. However, certain experimental initiatives have produced promising results (e.g. taste aversion in baboons: [18]; bees/chilli as deterrents to elephants: [19], [20]), but may require substantial funds (e.g. electric fences triggered by infra-red cameras: [21]). For behavioural-based management including conflict mitigation schemes, it is essential to have a good understanding of a species' ecological response to agricultural landscapes; past research demonstrates that ignoring behavioural data can lead to failure of management programs [22].

### Understanding species-wide patterns of crop feeding

The extent to which wild animals consume cultivars will depend on a variety of species-specific traits (e.g. ecological flexibility [23], mode of locomotion), as well as age/sex of individuals [24]. Consequently, it often makes sense for conflict mitigation strategies to focus on particular species [25]. Nevertheless, crop feeding within a species is further influenced by a complex interaction between local climatic (rainfall), ecological (particular crops grown, crop maturity, wild food availability) and anthropogenic factors (level of farm protection, proximity of fields to forest, human impact on natural food sources) [26], [27]. Although this suggests that mitigation strategies must be site-specific, some important generalisations can be made by examining patterns in crop feeding across habitats and populations. For example, if a crop is consumed by one population of a species, the same crop has the *potential* to be eaten by conspecific populations elsewhere if available. Because human-modified habitats are dynamic, the diets of wildlife inhabiting such environments often reveal some fluidity [5], [7]. Thus, if a crop is not currently exploited by a particular population, but conspecifics elsewhere consume the same crop habitually, it might be incorporated into the feeding repertoire in future years. Likewise, if certain crops are raided by multiple populations of a species across a variety of habitats – while others are consistently ignored despite being frequently available – this species-wide information enables identification of potential ‘high’ and ‘low’ conflict crops. Such data can be used to predict conflicts likely to occur under certain land-use change conditions and with the introduction of novel crops. Understanding which crops are potentially attractive or unattractive to a protected crop-raiding animal therefore has value for informing agricultural policy, for developing appropriate preventative measures, and for better directing resources for farm protection. This information is also critical for conservation initiatives that must consider human–wildlife conflict issues even where projects have other specified conservation objectives (e.g. to develop a corridor linking fragmented wildlife populations).

Chimpanzees provide an ideal model to explore species-wide patterns of cultivar consumption. While classified as endangered [28], this species occurs in areas of anthropogenic influence throughout tropical Africa [29]–[32]. Studies to date suggest that where chimpanzee home ranges encompass or border agricultural areas the apes incorporate cultivated crops into their diet to varying degrees [33]. Chimpanzee diets are diverse. Individual populations consume parts of up to 200 plant species including fruits, leaves, pith, flowers, and bark [34]–[37]. Nevertheless, chimpanzee diets are consistently dominated by ripe fruit, irrespective of habitat (e.g. dense lowland rainforest, dry savanna woodland or montane forest). Thus, unlike many crop-raiding species in Africa that are generalist feeders, including baboons, elephants, and vervets (*Chlorocebus* spp.), chimpanzees are ripe fruit specialists [38].

Comprehensive analyses of cultivar selection by protected large mammal species are lacking. Here, we review the literature to understand patterns of cultivar consumption by chimpanzees and consider cultivar feeding in the context of species-specific dietary strategies. We test the following hypotheses:

1. As studies indicate chimpanzees can adapt to human-influenced habitats, chimpanzees will consume cultivars throughout their geographical range.
2. As chimpanzees exhibit ecological and behavioural flexibility and a varied diet, they will consume an array of cultivars; populations at sites with a high exposure to agriculture will consume a greater range of cultivars than those with less exposure.
3. If chimpanzee crop consumption parallels wild feeding behaviour, chimpanzees will mainly target cultivated sugar fruits.
4. If a general relationship exists between overall crop availability and crop consumption by chimpanzees, crops that are most widely cultivated in chimpanzee range countries will be exploited at the greatest proportion of sites.

We show how these data can be used to provide practical information for on-the-ground stakeholders (i.e. farmers, wildlife managers, and conservation and agricultural extension practitioners) to help mitigate human–wildlife conflicts. We achieve this by integrating an understanding of chimpanzee crop utilisation with data on crop production in chimpanzee range countries in tropical Africa, together with the economic value (i.e. subsistence or commercial) of different crops to farmers. This enables characterisation of crops according to their potential to cause conflict.

## Methods

### Searching and Selection

In this article the terms ‘cultivar’ and ‘crop’ are used interchangeably. We defined a cultivar as “an assemblage of plants that (a) has been selected for a particular character or combination of characters, (b) is distinct, uniform and stable in these characters, and (c) when propagated by appropriate means, retains those characters” ([39] pp.6). We conducted a comprehensive literature search for records of cultivar feeding by wild chimpanzees. Both of us have studied wild chimpanzee diets in anthropogenic habitats [40], [41] and are familiar with the general literature on chimpanzee feeding ecology. Therefore, we first checked our own extensive collections of material pertaining to chimpanzee diet, including unpublished reports and theses. We then searched for additional records using Google Scholar and the Web of Science. We reviewed all manuscripts that referred to (i) chimpanzee plant feeding ecology (we did not consider material dealing predominantly or exclusively with faunivory), and (ii) chimpanzee use of anthropogenic environments, and extracted all data on crop consumption. Any reference in these articles to crop feeding which originated from additional published and unpublished sources was located, reviewed, and relevant data extracted. We excluded records of crop feeding if no information about specific crops eaten was provided. Where information about cultivars eaten by chimpanzees at a particular site came from >1 source, we examined them for agreement and retained the most authoritative source only, except where multiple sources together accounted for the range of crops recorded eaten. This resulted in a total of 33 sources, spanning 1931–2011.

We aimed to identify which cultivars are eaten rather than the manner in which they are obtained, since records did not always distinguish crop-raiding from crop feeding from abandoned sources, naturalised specimens or provisioned items. Nevertheless, most sources present chimpanzee crop feeding within the general context of crop-raiding (i.e. taking food that local people view as belonging to them). Therefore we assume that all consumed crops are potentially raided. We excluded feeding records for predominantly wild or naturalised plants that are occasionally cultivated or tended to by people. These included oil-palm (*Elaeis guineensis*), baobabs (*Adansonia digitata*), tamarind (*Tamarindus indica*), *Raphia* palms and figs (*Ficus* spp.).

The following data collection methods were used by authors to record cultivar feeding (including cultivar species and part eaten): (1) direct observation, (2) faecal analysis, (3) examination of feeding traces, (4) local people's reports, and (5) unspecified methods. We considered records made using methods 1–3 to constitute reliable evidence of cultivar feeding at a given site (‘confirmed foods’). Records based on local reports or an unspecified method indicated that a particular cultivar was potentially eaten at a site, but were not considered evidence of consumption (‘unconfirmed foods’). Local people's reports about which crops are eaten by particular wildlife are often accurate, but are inherently subjective [41], [42]. Reports may be unreliable due to misidentification of raiding species or if information imparted about crops eaten is imprecise or false (for example, by individuals seeking compensation or wishing to emphasise crop damage sustained). The sum of confirmed and unconfirmed crops constituted the full range of cultivated foods recorded eaten by chimpanzees (‘recorded foods’). Few articles included data on proportion of feeding time devoted to specific crops, so analyses were restricted to counts.

Twenty-four site records concerned single chimpanzee groups (‘communities’) or local populations, but three nationwide surveys were also included. Whereas many site records concerned information about crop consumption by single chimpanzee communities, other records were for wider areas (e.g. a national park) and were known or suspected to involve >1 chimpanzee community. Two (of three) nationwide surveys [43], [44] contained information about chimpanzee cultivar consumption from numerous localities (e.g. villages). It was not possible to determine if records from localities clustered in geographical space concerned one of more chimpanzee communities. Therefore nationwide surveys were treated as single site records, unless stated otherwise.

We categorised sites according to level of agricultural exposure: ‘High’ exposure applies to chimpanzees in fragmented landscape mosaics that include extensive areas of farmland and human settlements in addition to typically-small areas of uncultivated habitat. ‘Medium’ exposure applies to chimpanzees that range within a large expanse of uncultivated habitat such as a forest reserve or national park but whose territory borders farmland. ‘Low’ exposure applies to chimpanzees that range wholly within a large expanse of uncultivated habitat such as a rainforest. Such chimpanzees have limited access to cultivars due to low-level encroachment or the presence of abandoned gardens or settlements, or naturalised specimens.

### Cultivar availability

The presence of cultivars not consumed by chimpanzees was rarely noted by authors, so the full range of crops available at each site was unknown. Furthermore, records for total crop area per site were not available. Such data requires detailed site-specific local knowledge of chimpanzee ranging patterns in combination with human agricultural planting practices that often exhibit inter- and intra-annual variation. It was therefore beyond the scope of this study to use local availability of crops per site as a measure of availability. Instead, we obtained a general measure of availability for crops grown in chimpanzee range countries using data on area harvested per country from the Food and Agriculture Organization of the United Nations [45]. Although records of chimpanzee cultivar consumption date back to the 1930s, we used the most recent FAO census data from 2009 to understand how current agricultural activities might impact present and future human–chimpanzee conflicts. The FAO data are derived from nationwide surveys conducted by each respective country and have certain limitations. In particular, most census data are likely restricted to commercial agricultural activities, omitting small-scale subsistence farming [46]. This is further indicated by the fact that certain domestic crops that are widely farmed in tropical Africa have very low values for area harvested (see below). Therefore, we assumed that crops harvested in areas greater than 1000 ha (10 km^2^) per country are likely commercial cultivars (i.e. cash crops). We considered commercial cultivars to be both important and widespread (‘important widespread commercial crops’) if they were harvested in areas greater than 1000 ha in >50% of chimpanzee range countries (i.e. in ≥11 of the 21 countries in which chimpanzees currently occur; [47]). Our approach is necessarily broad – there is likely to be considerable localised geographical and temporal variation in crops grown per country – but more specific data on crop production are lacking. The Food and Agriculture Organization of the United Nations [45] provide data on area harvested for crops grown in chimpanzee range countries. While data are available for most individual crops, FAO presents summed data for certain groups of crops. In Table S1 and related analyses we excluded data for broad categories such as ‘fresh fruit’, to avoid replication of individual fruits, but we included one crop group, bean, as some are recorded eaten by chimpanzees. Mango and guava are combined by FAO, but we separated them since these are confirmed chimpanzee foods, assuming that each is harvested in an equivalent area in the same number of countries. We did the same for lemon and lime because lemon is also a confirmed chimpanzee food.

### Conflict Classification

Many food crops are also grown for subsistence purposes. Subsistence cultivars were categorised as human ‘staples’ (i.e. eaten regularly and in such quantities as to constitute an important part of the diet and supply a major proportion of energy and nutrient needs), or ‘non-staples’, such as domestic fruits and spices. We categorised crops according to their likelihood to cause human–chimpanzee conflict. ‘High conflict’ applies to important widespread commercial (IWC) crops and/or staple subsistence crops that were consumed at ≥25% of sites at which chimpanzee crop feeding was recorded. This cut-off enables identification of crops eaten by numerous populations (i.e. its consumption is not peculiar to a small number of communities), and is therefore appropriate for projecting the likelihood that the same crop would be consumed by other chimpanzee communities if available. ‘Potentially high conflict’ applies to non-staple subsistence crops recorded eaten at ≥25% of sites. ‘Low conflict’ applies to non-staple subsistence crops and/or non-IWC crops (harvested in >1000 ha in less than 11 countries) for which there were no records of chimpanzee consumption. Crops assumed to be inedible raw due to toxic compounds or extreme spiciness were always considered ‘low conflict’. ‘Potentially low conflict’ applies to IWC and/or staple crops not recorded eaten by chimpanzees, or else the part eaten is unimportant to humans and its consumption does little damage to the plant. We excluded highly palatable crops from the ‘low conflict’ list (i.e. those very similar in taste to frequently consumed wild or cultivated foods) for which an absence of feeding records by chimpanzees likely reflects low exposure. Crops that were not classified as high or low conflict according to these criteria were considered ‘intermediate’, accepting that consumption by chimpanzees might create conflict under certain local conditions.

As noted above, while the FAO data provide a good general measure of crops that are available to varying extents within the chimpanzees' geographical range, they do not yield data on exact crop availability at the specific study sites included in this analysis. Consequently, conflict definitions – which are derived partially from FAO data – are intended as a guide only.

### Data Synthesis and Analysis

Data were analysed using SPSS version 19. We used non-parametric tests because data were non-normally distributed. Although meta-analyses are often used to test large collections of results [48], all data obtained on the dependent variable – number of crops consumed per site – were counts, in some cases summed from >1 source, making meta-analysis invalid. In addition, few studies were specifically concerned with recording all crops eaten by a particular community/population. To test whether exposure level affected the number of confirmed cultivars eaten per site (n = 24 sites), we performed a Kruskal–Wallis test, and used Post-hoc Mann–Whitney pair-wise comparisons (with Bonferonni correction) to reveal differences between exposure levels. To test for agreement between data collection methods in individual crops and crop parts recorded eaten, we used Spearman rank correlations to assess the relationship between (i) the proportion of confirmed and unconfirmed crop foods in different plant part categories, and (ii) the number of confirmed and unconfirmed site records for each crop. To assess whether chimpanzee crop feeding follows a species-typical pattern, we employed a Spearman correlation to test the accordance between the proportion of confirmed crop foods in different plant part categories (fruit, pith, leaf, seed, flower, bark and other) and the mean proportion per category of the total plant food diet at 10 chimpanzee study sites (using data from Morgan & Sanz [49]). The 10 study sites are: Assirik, Belinga, Bossou, Gombe, Goualougo, Kahuzi, Lópe, Mahale, Ndoki and Semliki. We conducted a Mann–Whitney test to determine whether IWC crops were consumed at a greater % sites than more sparsely distributed crops. For fruit crops and non-fruit crops separately, we tested the relationship between availability (indexed as the number of range countries with area harvested >1000 ha) and % sites at which each crop was eaten with Spearman correlations. Because humans and chimpanzees might utilise different parts of the same crop (e.g. cashew fruit: [50]), we used Fisher's exact test to determine if a relationship exists between the conflict level associated with a particular crop and the crop part utilised by humans. All hypotheses considered were two-tailed and tested at α = 0.05.

## Results

### Flow of Included Studies

**Figure 1:**
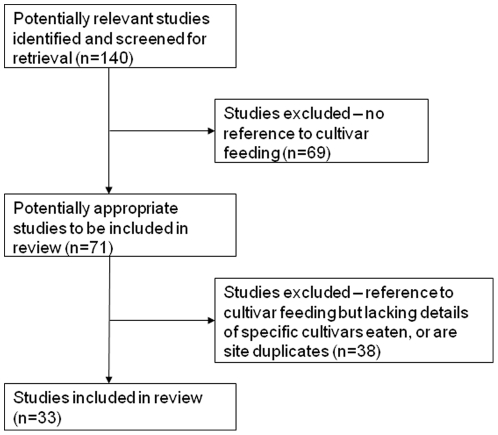
The flow of studies included in the analysis.

Figure 1 shows the flow of studies included in the analysis.

### Do Chimpanzees Consume Crops Throughout their Geographical Range?

**Figure 2:**
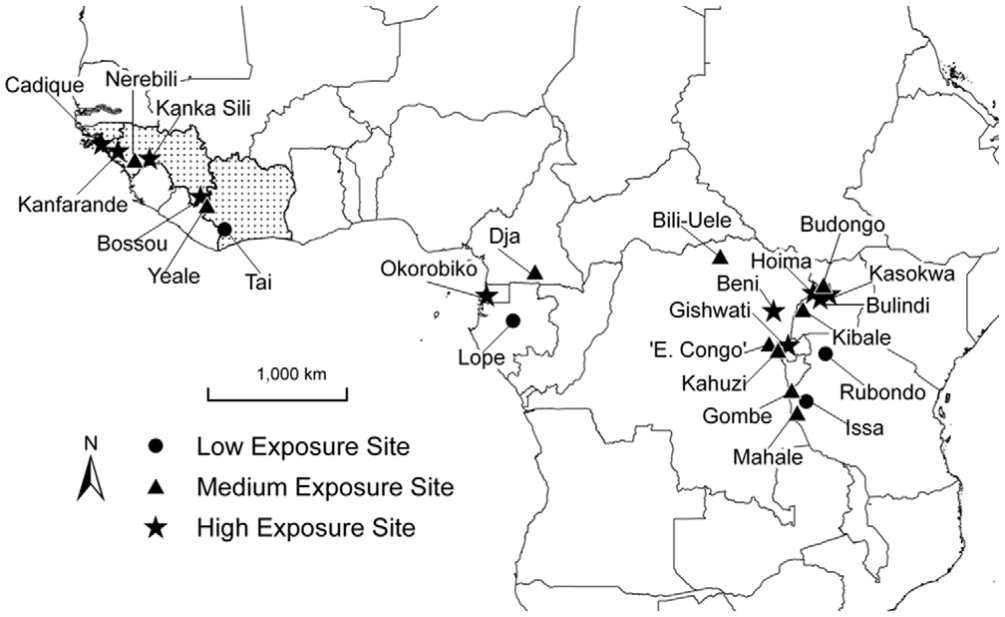
Map showing the locations of sites where chimpanzees were recorded to consume cultivars. Sites were classified as high, medium or low exposure to agriculture. Ten countries are represented: *West Africa* – Guinea-Bissau, Guinea, Cote d'Ivoire; *Central Africa* – Gabon, Equatorial Guinea, Cameroon, Democratic Republic of Congo; *East Africa* – Uganda, Rwanda, Tanzania. See Table 1 for site names. Nationwide surveys recorded chimpanzee crop feeding at multiple localities in Guinea-Bissau, Guinea and Cote d'Ivoire (dotted countries).

Records of cultivar consumption by chimpanzees came from 27 sites, of which three were nationwide survey reports that include records from multiple localities. The sites span 10 countries in East, West and Central Africa (Figure 2). Countries with the most site records were Guinea and Uganda (5 each), followed by Tanzania and Democratic Republic of Congo (DRC) (4 each). Three of the four commonly recognised chimpanzee subspecies were represented: *Pan t. verus*, *Pan t. troglodytes* and *Pan t. schweinfurthii* in western, central and eastern Africa, respectively (the exception is the little-studied *Pan t. elliotti* in Nigeria–Cameroon). Excluding nationwide surveys, 10 sites were classified as high exposure to agriculture, 10 as medium exposure and 4 as low exposure (Figure 2). Cultivar consumption was recorded from all major habitats where chimpanzees occur, including lowland rainforest, mid-altitude forest, montane forest and savanna–woodland.

### Do Chimpanzees Eat an Array of Cultivars?

A total of 34 plant parts from 24 species of cultivar were confirmed eaten by chimpanzees, while an additional 17 plant parts and 12 species were unconfirmed (Table 1). Inclusion of these species brings the total number of cultivated plant parts and species recorded eaten to 51 and 36, respectively. The number of different cultivars eaten varied among sites (n = 24, excluding nationwide surveys). The median number of confirmed cultivars per site was one (range: 0–14) and the median number of recorded cultivars was three (range: 1–26). While there was no significant effect of exposure level on the number of confirmed cultivars eaten per site (Kruskal–Wallis test, H = 3.012, df = 2, p = 0.22), a significant effect was found for all recorded crops (H = 7.475, p = 0.02). Post-hoc Mann–Whitney pair-wise comparisons revealed no difference between medium and high exposure sites (p = 0.48); however, fewer crops were eaten at low exposure sites compared to medium (p = 0.008) and high sites (p = 0.02; Bonferroni correction: p = 0.017). A single cultivar was recorded eaten at each low exposure site.

**Table 1:** List of cultivars recorded in diets of wild chimpanzees and part(s) eaten.

Crop	Part Eaten^1^	Study Sites^2^	No. Sites
		Confirmed (Unconfirmed)	Confirmed (All records)^3^
Avocado (*Persea americana*)	F	(Bos, Bul)	0 (2)
	L	Bos	1
Banana (*Musa* spp.)^4^	F	Ben, Bos, Bul, Gom, Kib, Mah, Oko (Bil, Cad, Con, Gui, Hoi)	7 (12)
	P	Bos, Bul, Kib, Mah, Ner, Oko (Con)	6 (7)
	L	Mah	1
	Un	(Dja, Ivo, Kah)	0 (3)
Butter bean (*Phaseolus lunatus*)	L	(Bos)	0 (1)
	S	(Bos)	0 (1)
Cantaloupe (*Cucumis melo*)	F	(Bos)	0 (1)
Cashew (*Anacardium occidentale*)	F	Cad (Bos)	1 (2)
Cassava (*Manihot esculenta*)	Fl	(Bos)	0 (1)
	T	Bos, Oko (Gui, Hoi, Yea)	2 (5)
Cocoa (*Theobroma cacao*)	F	Bos, Bul, Dja (Hoi, Ivo, Taï, Yea)	3 (7)
Coconut (*Cocos nucifera*)	F	(Bos)	0 (1)
Coffee (*Coffea* sp.)	Un	(Ivo)	0 (1)
Cow pea (*Vigna unguiculata*)	S	Cad	1
Cucumber (*Cucumis sativus*)	Un	(Dja)	0 (1)
Grapefruit (*Citrus paradisi*)	F	Bos, Sil	2
Guava (*Psidium guajava*)	F	Bud, Bul, Mah (Bos, Cad)	3 (5)
	L	(Bos)	0 (1)
Jackfruit (*Artocarpus heterophyllus*)	F	Bul, Hoi	2
Lemon (*Citrus limon*)	F	Mah, Rub (Cad)	2 (3)
Maize (*Zea mays*)	F	Bos, Bud, Gui, Kib (Yea)	4 (5)
	P	Gis, Mah (Bul)	2 (3)
	Un	(Bis, Hoi, Ivo, Kah)	0 (4)
Mandarin (*Citrus reticulata*)	F	Bos (Cad)	1 (2)
Mango (*Mangifera indica*)	F	Bos, Bud, Bul, Cad, Gom, Gui, Iss, Kas, Lop, Mah (Hoi, Kan)	10 (12)
Millet (unknown sp.)	Un	(Gui, Sil)	0 (2)
Okra (*Abelmoschus esculentus*)	F	Bos	1
	L	Bos	1
	Fl	Bos	1
Orange (*Citrus sinensis*)	F	Bos, Bul, Cad, Gui (Yea)	4 (5)
Papaya (*Carica papaya*)	F	Ben, Bil, Bos, Bud, Bul, Cad, Kas (Gui, Hoi, Ivo, Yea)	7 (11)
	P	Bos, Cad	2
	L	Bos, Cad	2
	B	Bos	1
	W	Bos	1
Passion fruit (*Passiflora* sp.)	F	Bul, Kas, Kib	3
Peanut (*Arachis hypogaea*)	S	(Bos)	0 (1)
Pigeon pea (*Cajanus cajan*)	S	Mah (Bos)	1 (2)
Pineapple (*Ananas comosus*)	F	Bos (Bul, Gui, Hoi, Ivo, Yea)	1 (6)
	P	Bos	1
Pumpkin (*Cucurbita* sp.)	F	(Bos, Bul, Hoi)	0 (3)
Rice (*Oryza* sp.)	P	Bos (Ivo, Sil, Yea)	1 (4)
Sorghum (*Sorghum bicolor*)	P	Mah (Hoi)	1 (2)
Soursop (*Annona muricata*)	F	(Bos)	0 (1)
Sugarcane (*Saccharum officinarum*)	P	Bil, Bos, Bud, Bul, Kas, Kib, Mah, Oko (Bis, Con, Gui, Hoi, Ivo)	8 (13)
Sweet potato (*Ipomoea batatas*)	T	(Bos)	0 (1)
Tamarillo (*Solanum betaceum*)	F	Bul	1
Tea (*Camellia sinensis*)	Fl	(Gui)	0 (1)
Tomato (*Solanum lycopersicum*)	F	(Bos, Bul)	0 (2)
Yam (*Dioscorea* sp.)	P	Bul	1
	T	(Bos)	0 (1)

1 Part Eaten: F = fruit, P = pith, L = leaf, S = seed, Fl = flower, T = tuber, B = bark, W = wood, Un = unspecified;

2 Study Sites (**+** = site record known or likely to concern ≥1 chimpanzee community; # = record is a nationwide survey comprising multiple localities): (a) *Pan t. schweinfurthii*: Ben = Beni [56], [57]; Bil = Bili-Uele**^+^** [58]; Bud = Budongo**^+^** [37]; Bul = Bulindi [41]; Con="East Congo”**^+^** [59]; Gis = Gishwati [60]; Gom = Gombe [35]; Hoi = Hoima District**^+^** [31]; Iss = Issa [61]; Kah = Kahuzi-Biega**^+^** [62]; Kas = Kasokwa [63], [64]; Kib = Kibale**^+^** [26], [65]; Mah = Mahale**^+^** [34], [66]; (b) *Pan t. troglodytes*: Dja = Dja**^+^** [67], [68]; Lop = Lópe**^+^** [36]; Oko = Okorobikó Mtns [69]; (c) *Pan t. verus*: Bis = Guinea-Bissau^#^ [70]; Bos = Bossou [30], [40]; Cad = Caiquene & Cadique [32]; Gui = Guinea^#^ [44]; Ivo = Côte d'Ivoire^#^ [43]; Kan = Kanfarande [71]; Ner = Nérébili [72]; Sil = Kanka Sili [29]; Taï = Taï [73]; Yea = Yeale, Mt Nimba (Granier, in [33]); Rub = Rubondo [74].

3 Sites at which the cultivar was *confirmed* eaten via direct observation, faecal analysis and/or feeding traces, are distinguished from *unconfirmed* sites at which consumption was recorded via local reports or an unspecified method. ‘All records’ is the sum of confirmed and unconfirmed sites.

4 Includes both plantain and sweet bananas.

### Do Chimpanzees Mainly Target Sugar Fruit Crops?

**Figure 3:**
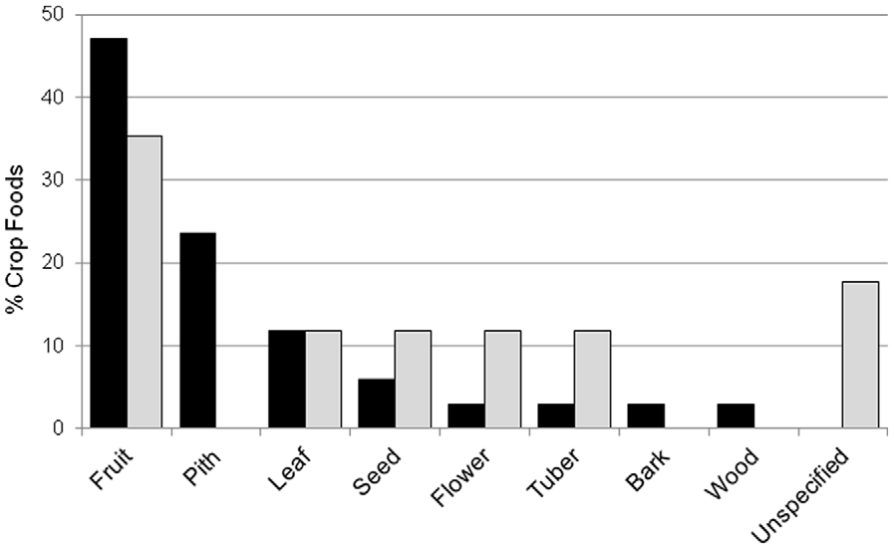
Profile of cultivated plant parts recorded eaten by chimpanzees. The number of food items in each part category is shown as a percentage of all confirmed (black bars, n = 34) and unconfirmed (grey bars, n = 17) cultivated food items. The proportion of confirmed and unconfirmed crop foods per category is uncorrelated (Spearman's rank correlation: r_s_ = 0.074, n = 9, p = 0.85).

**Figure 4:**
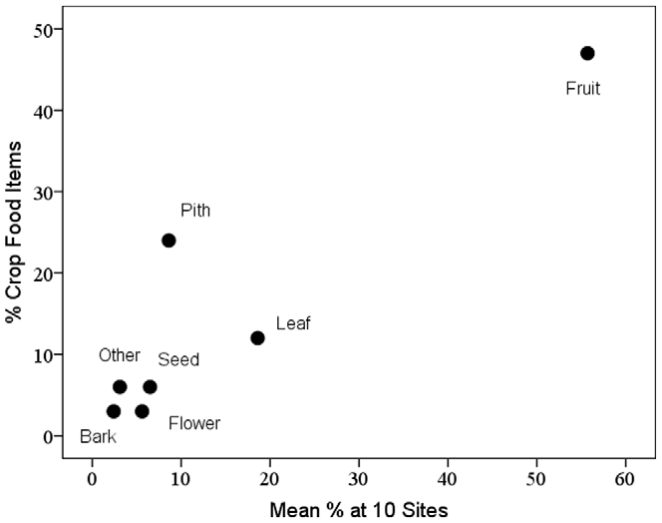
Parallels between crop consumption and wild food consumption. Relationship between the percentage of confirmed crop foods in different plant part categories and mean percentage per category of all plant foods at 10 chimpanzee study sites. ‘Other’ plant parts include resin, tuber and wood.

Eight different crop plant parts were recorded eaten: fruits, piths, leaves, seeds, flowers, tubers, bark and wood, in addition to unspecified parts. Fruits dominate the list of cultivated food items, accounting for 16 of 34 (47%) confirmed items (Figure 3). Aside from piths, other plant parts from cultivated species were rarely confirmed as food. Conversely, a greater proportion of unconfirmed crop foods were seeds, flowers, and tubers, or from an unspecified part of the plant (Figure 3). The composition of crop food parts followed a species-typical pattern: the proportion of confirmed crop foods in different plant part categories was positively correlated with the mean proportion per category of the total plant food diet at 10 chimpanzee study sites (r_s_ = 0.873, n = 7, p = 0.01; Figure 4).

For each crop confirmed eaten, the number of unconfirmed site records was strongly correlated with the number of confirmed site records (r_s_ = 0.510, n = 34, p = 0.002), indicating agreement between data collection methods in the crops commonly exploited by chimpanzees. Sugar fruits were widely eaten (mango, papaya, banana) as were three pith foods (sugarcane, banana, maize) (Table 2). When all records are considered, banana was the most widely consumed crop, followed by sugarcane, mango, maize, papaya and cocoa.

**Table 2:** Cultivars most commonly recorded eaten by chimpanzees.

Crop^1^	Part Eaten^2^	No. Sites (n = 27)	% Sites^3^
		Confirmed (All records)	
Mango	Fruit	10 (12)	37.0–44.4%
Banana	Fruit, Pith	8 (16)	29.6–59.3%
Sugarcane	Pith	8 (13)	29.6–48.1%
Papaya	Fruit	7 (11)	25.9–40.1%
Maize	Fruit, Pith	6 (12)	22.2–44.4%
Cocoa	Fruit	3 (7)	11.1–25.9%

1 Crops listed are those recorded eaten at ≥25% of sites.

2 Banana fruit- and pith-eating, and maize fruit- and pith-eating, were not distinguished because part(s) eaten was not specified in some reports. For these crops the number of site records for each specified part is shown in Table 1.

3 Percentage ranges indicate the % sites at which each crop was confirmed eaten (lower value) and recorded eaten (confirmed and unconfirmed combined: higher value).

### Are Widespread Cultivars Most Commonly Targeted?

Of the 70 crops recorded eaten by chimpanzees and/or harvested in areas greater than 1000 ha in ≥1 chimpanzee range country (Table S1 for the supporting information table), 25 were IWC crops. Of these, 80% were also known subsistence crops (20 of 25), of which 12 (48%) were staple foods. While only 48% of IWC crops were confirmed chimpanzee foods (12 of 25), the figure rises to 76% (19 of 25) if unconfirmed records are included. IWC were consumed at a significantly greater % sites compared to more sparsely distributed crops (<11 countries with area harvested >1000 ha), although effect sizes were small (*confirmed crops*: Mann–Whitney test: U = 346, z = −2.095, p = 0.035, *r* = −0.033; *all recorded crops*: U = 234, z = −3.589, p<0.001, *r* = −0.057).

For all listed fruit crops (including nuts), there was no significant correlation between number of range countries (with area harvested >1000 ha) and % sites at which each crop was confirmed eaten (r_s_ = 0.311, n = 16, p = 0.24), but the correlation was significant for all records (r_s_ = 0.527, p = 0.036). For non-fruit crops, there was a significant correlation between the number of range countries and % sites at which the crop was confirmed (r_s_ = 0.485, n = 47, p<0.001) and recorded (r_s_ = 0.641, p<0.001) eaten.

### Do Crops Vary in their Likelihood to Cause Conflict?

Five crops were classified as ‘high conflict’: banana, sugarcane, maize, mango and cocoa (Table S1). A further two crops, papaya and oil-palm, were regarded as ‘potentially high conflict’. Twenty crops were considered ‘low conflict’, ten were ‘potentially low conflict’, and the remaining 33 were ‘intermediate’. There was a significant association between conflict level and the crop part utilised by humans (Fisher's exact test: p<0.001); 86% (6 of 7) of high or potentially high conflict crops were fruits compared with just 13% (4 of 30) of low or potentially low conflict crops, which were mostly seeds (30%, 9 of 30) or underground storage organs (23%, 7 of 30).

## Discussion

The survey revealed that chimpanzees consume cultivars across their geographic range in equatorial Africa, especially in Guinea in West Africa, and Uganda, Tanzania and DRC in East Africa. This probably reflects the fact that chimpanzees have been studied at several sites in each of these countries. However, another factor may be that apes and other nonhuman primates are not traditionally hunted for meat in Uganda, Tanzania and parts of Guinea [47], [51], thus enabling chimpanzees to persist in areas of agricultural expansion and high human population density – a scenario unlikely to emerge in regions where apes are heavily hunted. We found no crop feeding records for *P.t. elliotti* in Nigeria–Cameroon, but this may reflect a paucity of data rather than sub-species differences in feeding behaviour or exposure to agriculture.

Chimpanzees consumed up to 36 crop species – an unexpectedly diverse array given that chimpanzees are not considered opportunist feeders, unlike more ‘typical’ crop-raiding wildlife (e.g. baboons [7]; elephants [5]). As predicted, populations with greater exposure to agriculture consumed more crops than those with low exposure. At ‘low exposure’ sites, a single record of cultivar feeding may have involved naturalised specimens (e.g. mango at Lopé and Issa; lemon at Rubondo). Such populations seem to have few opportunities to raid crops. The greatest range of crops eaten was recorded at Bossou and Bulindi (Table 1), both heavily disturbed forest–farm mosaics where chimpanzees have a very high exposure to crops. However, researchers working at these sites have specifically considered the issue of chimpanzee crop feeding, including the range of items raided [40], [41]. That no difference was found between the number of crops eaten at high and medium exposure sites probably reflects the fact that comprehensive studies of crop feeding at high exposure sites are few. Nevertheless, crop consumption at lesser-impacted sites may be under-reported: cultivar feeding is often viewed as an ‘unnatural’ food habit, and thus unimportant or distinct from ‘natural’ feeding [52]. We expect more is known about chimpanzee crop-raiding, at sites included in this survey and at additional sites, but data are unpublished.

Although chimpanzees utilise various crop parts for food, they show a strong preference for sugar fruits, thus conforming to a species-typical pattern. Nevertheless, they also eat a range of cultivated piths, most notably sugarcane. Other crop parts such as leaves, seeds, flowers, tubers, bark and wood are exploited less often. The representation of fruits was higher in confirmed crop foods than unconfirmed foods. Fruit can be over-represented in dietary studies that rely on faecal analysis because non-fruit plant parts are seldom identifiable macroscopically in faeces [36]. Of 34 confirmed crop foods, 30 (88.2%) were recorded from direct observations at ≥1 sites while four others were recorded only from feeding traces. All crops recorded in chimpanzee faeces at particular sites were also observed eaten elsewhere. Therefore, the discordance between confirmed and unconfirmed crop parts does not reflect a fruit-bias in confirmed foods arising from inclusion of faecal evidence. The greater proportion of unconfirmed crop food items that were seeds, flowers or tubers might imply that local people's reports over-estimate non-fruit raiding by chimpanzees. Local reports of crop losses may be biased towards important commercial or staple subsistence crops; low-level raiding of domestic fruits may be tolerated in some situations [41].

**Figure 5:**
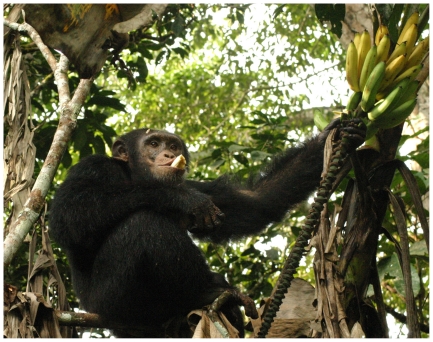
An adult male chimpanzee at Bossou in Guinea feeding on banana fruit.

The crops listed in Table 2 appear particularly attractive to chimpanzees whenever they are accessible. These most commonly consumed cultivars include predominantly domestic fruits (e.g. papaya, mango), but some are staples (maize, banana; see Figure 5) and others cash crops (e.g. sugarcane, cocoa). As predicted, widely cultivated crops were eaten at more sites than less widely distributed crops. Yet certain widely available cultivars were never or infrequently exploited. For example, cassava is farmed throughout all 21 chimpanzee range countries, but was not widely eaten. This suggests a degree of selectivity in crop choice among different chimpanzee populations. Similarly, while both the pith and fruit of banana is typically consumed at the same site (see Table 1), chimpanzees have not yet been confirmed eating both parts of maize. Food selection can vary between populations of the same species, particularly in primates including chimpanzees, and certain foods eaten by one population may be ignored by another despite being available [53]. The possible existence of different crop feeding traditions among chimpanzee populations warrants further investigation. Strong observational evidence indicates chimpanzees routinely ignore certain crop species (mainly non-fruits). This suggests they make *choices* about what crops to eat [40], [41].

Nationwide surveys of Côte d'Ivoire and Guinea demonstrate that certain crops that are widely farmed in particular countries, but less so in others, are heavily exploited by chimpanzees in those regions. For example, chimpanzee damage to cocoa plantations was recorded at 35% of 125 villages where chimpanzee presence was confirmed throughout the cocoa-growing forested region of Côte d'Ivoire – more than for any other recorded crop [43]. In contrast, in neighbouring Guinea – where cocoa is not widely farmed – chimpanzees were recorded to raid oranges at 32% of 74 sites where apes were not reported absent; cocoa raiding was not recorded [44]. To test whether the proportion of each crop in a particular chimpanzee population's diet is dependent on its local availability requires detailed site-specific data. As yet such data are unavailable, but future studies should examine this issue further.

### How do These Findings Help Address Human–Wildlife Conflict?

Conflict mitigation strategies that target problematic wildlife behaviours such as crop-raiding are notoriously difficult to develop for large-bodied, cognitively complex species, and require a good understanding of species' ecological flexibility. When species have protected status (e.g. all great apes), theoretically problem animals should only be deterred, translocated or tolerated, thus proactive management is required. In human-dominated landscapes protection of small areas of habitat is alone unlikely to be a sufficient conservation strategy if wildlife require wide ranges and frequently leave the forest to crop-raid [27], [31]. Effective long-term strategies require a combination of approaches that target wildlife behaviour, protect habitat, and increase local people's tolerance and secure their livelihoods [54]. Understanding species-wide crop feeding behaviour in relation to human agricultural activities – and combined with an understanding of crop-raiding behaviour of other sympatric wildlife – has enormous potential for informing on-the-ground stakeholders about cultivars that have the potential to cause or reduce human–wildlife conflict.

Conflicts associated with chimpanzee raiding of the crops categorised as ‘high conflict’ (Table S1) have been documented [e.g. 31], [33], [37], [41], [44]. The results of this study imply that conflicts might be reduced or prevented if farmers avoid planting crops identified as ‘high conflict’ or ‘potentially high conflict’ in very close proximity to chimpanzee habitat (e.g. along forest edges). Conversely, cultivars identified as ‘low conflict’ (including those that are inedible when raw) or ‘potentially low conflict’ are unlikely to attract chimpanzees and could potentially act as a buffer to other forms of land-use. In this respect, it is important that several widespread cash crops and staple food crops are seemingly seldom or never exploited by chimpanzees (Table S1). Our classifications are intended to apply to situations where crops are guarded rather than abandoned. Evidently, chimpanzees' feeding on a high-conflict crop such as mango or banana is unlikely to cause high levels of conflict if it is from an abandoned source. The dynamic nature of human–wildlife interactions and conflict must also be considered. Orange was not flagged as a high conflict crop, probably because it is not widely grown in some range countries. However, it is frequently raided by chimpanzees in certain regions where it is increasingly farmed commercially (e.g. in Guinea [40], [44]). Thus, where orange is grown predominantly as a cash-crop, rather than as a domestic fruit, chimpanzee raiding is predicted to cause high conflict.

However, the applicability of our findings for conflict management in forest–agricultural mosaics is constrained by several factors: (i) low conflict crops may be associated with increased conversion of forest habitat (e.g. tobacco in Uganda [31]; cashew in Guinea-Bissau [50]); (ii) crops that are unattractive to one species may be readily targeted by other sympatric wildlife; (iii) farmers' landholdings are frequently small in Africa, limiting choice in crop spatial arrangement; (iv) decisions regarding which crops to plant are determined chiefly by cultural, practical and/or economic factors; (v) highly mobile species, including chimpanzees, may travel several hundred metres across farmland to reach preferred cultivars [41]; thus buffer crops may be ineffective at preventing raids; and (vi) conflict associated with crop-raiding is exacerbated by aggressive behaviour directed at people during encounters [55]. This implies that any crop-feeding by large mammals will potentially cause conflict if it increases contact with people.

Clearly, wildlife and conservation managers must carefully consider crop characteristics before liaising with the agricultural sector and making land-use recommendations that concern crops. Further to their palatability to crop-raiding wildlife, the economic importance (e.g. commercial potential, profitability and sustainability) and physical characteristics (e.g. crop growing time; in addition, tall crops or dense orchards provide cover for raiding animals, enabling travel between agricultural areas) should be taken into account. Crop suitability must be assessed in terms of local people's requirements (e.g. nutritional value, storage capacity, preparation techniques, processing time required), and cultural factors (e.g. agricultural knowledge, food preferences and traditions). The utility of particular crops to reduce conflict must be balanced against their environmental impact. Different conflict issues (e.g. crop-raiding and aggressive interactions between people and large mammals) should not be considered in isolation, but should be addressed as part of an integrative management plan.

Finally, we urge researchers to accord crop consumption and selection by individual wildlife species greater importance. At few sites where mammals are studied are they entirely unexposed to agricultural plants. Thus, crop feeding should be considered within the context of the animal's ecological adaptation to its current environment. Further, with ongoing habitat conversion and land-use changes globally, increased exposure of wildlife to agriculture is unavoidable. If we are to fully understand the responses of endangered and ecologically specialised species to changing environments and contact with agriculture – and thus to develop effective management strategies to reduce conflict with people and safeguard the species' future – availability of quantitative data on cultivar consumption is of paramount importance.

## Supporting Information

Table S1 **List of crops cultivated in chimpanzee range countries and their potential to cause human–chimpanzee conflict.** Cultivars are listed if they are recorded eaten by wild chimpanzees at ≥1 site, and/or they are harvested in ≥1 range country in areas greater than 1000 ha, according to FAOSTAT (for 2009).
(PDF)
