# Supplementary material for: From Forest to Farm: Systematic Review of Cultivar Feeding by Chimpanzees – Management Implications for Wildlife in Anthropogenic Landscapes
Source: PLoS One. 2012 Apr 11;7(4):e33391. doi: 10.1371/journal.pone.0033391 (PMC3324475; doi:10.1371/journal.pone.0033391)
Supplement: Table S1 — List of crops cultivated in chimpanzee range countries and their potential to cause human–chimpanzee conflict. Cultivars are listed if they are recorded eaten by wild chimpanzees at ≥1 site, and/or they are harvested in ≥1 range country in areas greater than 1000 ha, according to FAOSTAT (for 2009). (PDF) [file pone.0033391.s001.pdf]

Table S1. List of crops cultivated in chimpanzee range countries and their potential to cause human–chimpanzee conflict.

Crop Type [1]	Crop [2]	Human Part [3]	No. Countries [4] (n = 21)	Subsistence Non-staple	Subsistence Staple	% site records [5]	Conflict [6]
**Cereals**	Barley *(Hordeum vulgare)*	G	2		x	0%	
	Maize *(Zea mays)*	F	19		x	22.2–44.4%	H
	Millet *(various spp.)*	G	18		x	0–7.4%	
	Rice *(Oryza sp.)*	G	19		x	3.7–14.8%	
	Sorghum *(Sorghum sp.)*	G	16		x	3.7–7.4%	
	Wheat *(Triticum sp.)*	G	9		x	0%	
**Vegetables & melons**	Aubergine *(Solanum melongena)*	F	4	x		0%	L
	Cabbage *(Brassica oleracea)*	L	5	x		0%	L
	Cantaloupe *(Cucumis spp.)*	F	0	x		0–3.7%	
	Carrot *(Daucus carota)*	U	2	x		0%	L
	Cucumber *(Cucumis sativus)*	F	3	x		0–3.7%	
	Okra *(Abelmoschus esculentus)*	F	5	x		3.70%	
	Onion *(Allium cepa)*	U	11	x		0%	
	Leek *(Allium ampeloprasum)*	P	1	x		0%	L
	Pumpkin *(Cucurbita sp)*	F	7	x		0–11.1%	
	Tomato *(Solanum lycopersicum)*	F	13	x		0–7.4%	
	Watermelon *(Citrullus lanatus)*	F	3	x		0%	p
**Fruits & nuts**	Avocado *(Persea americana)*	F	5	x		3.7–7.4%	
	Banana *(Musa spp.)*	F	20	x	x	29.6–59.3%	H
	Cashew *(Anacardium occidentale)*	S	9	x		3.7–7.4%	
	Coconut *(Cocos nucifera)*	F	11	x		0–3.7%	
	Date *(Phoenix dactylifera)*	F	1	x		0%	p
	Grape *(Vitis vinifera)*	F	1	x		0%	p

	Grapefruit *(Citrus paradisi)*	F	2	x		7.4%	
	Guava (*Psidium guajava)*	F	12	x		11.1–18.5%	
	Jackfruit *(Artocarpus heterophyllus)*	F	0	x		7.4%	
	Kola nut *(Cola sp.)*	S	5	x		0%	L
	Lemon *(Citrus limon)*	F	4	x		7.4–11.1%	
	Lime *(Citrus aurantifolia)*	F	4	x		0%	p
	Mandarin *(Citrus reticulata)*	F	0	x		3.7–7.4%	
	Mango *(Mangifera indica)*	F	12	x		37–44.4%	H
	Orange *(Citrus sinensis)*	F	10	x		14.8–18.5%	
	Papaya *(Carica papaya)*	F	4	x		25.9–40.7%	
	Passionfruit *(Passiflora sp.)*	F	0	x		11.1%	
	Peanut *(Arachis hypogaea)*	S	21	x		0–3.7%	
	Pineapple *(Ananas comosus)*	F	12	x		3.7–22.2%	
	Soursop *(Annona muricata)*	F	0	x		0–3.7%	
	Tamarillo *(Solanum betaceum)*	F	0	x		3.7%	
**Oilseed crops**	Castor bean *(Ricinus communis)*	S	4			0%	L +
	Oil palm *(Elaeis guineensis)*	F	17		x	–	
	Safflower *(Carthamus tinctorius)*	S	1	x		0%	L
	Sesame *(Sesamum indicum)*	S	13	x		0%	
	Shea nut *(Vitellaria paradoxa)*	S	5		x	0%	
	Soya bean *(Glycine max)*	S	10		x	0%	L +
	Sunflower *(Helianthus annuus)*	S	4			0%	L
**Root/tuber crops**	Cassava *(Manihot esculenta)*	U	21		x	7.4–18.5%	
	Potato *(Solanum tuberosum)*	U	11		x	0%	
	Sweet potato *(Ipomoea batatas)*	U	18		x	0–3.7%	
	Taro *(Colocasia esculenta)*	U	11		x	0%	L +
	Yam *(Dioscorea sp.)*	U	15		x	3.7–7.4%	

	Yautia *(Xanthosoma spp.)*	U	1		x	0%	L +
**Beverage & Spice crops**	Chilli/pepper *(Capsicum spp.)*	F	10	x		0%	L +
	Clove *(Syzygium aromaticum)*	F	1	x		0%	L +
	Cocoa *(Theobroma cacao)*	F	14			11.1–25.9%	H
	Coffee *(Coffea sp.)*	S	16			0–3.7%	
	Garlic *(Allium sativum)*	U	1	x		0%	L
	Ginger *(Zingiber officinale)*	U	2	x		0%	L
	Nutmeg/cardamom *(various spp.)*	S	1	x		0%	L
	Pepper *(Piper spp.)*	F	3	x		0%	L +
	Tea *(Camellia sinensis)*	L	6			0–3.7%	
**Leguminous crops**	Bean *(various spp.)*	S	13		x	0–3.7%	
	Cow pea *(Vigna unguiculata)*	S	9		x	3.70%	
	Pigeon pea *(Cajanus cajan)*	S	4		x	3.7–7.4%	
**Sugar crops**	Sugar cane *(Saccharum officinarum)*	P	19			29.6–48.1%	H
**Other crops**	Abacá *(Musa textilis)*	Fib	1			0%	p
	Cotton *(Gossypium sp.)*	Fib	15			0%	
	Pyrethrum *(Chrysanthemum spp.)*	Fl	2			0%	L
	Rubber *(Hevea brasiliensis)*	La	10			0%	L
	Sisal *(Agave sisalana)*	Fib	2			0%	L
	Tobacco *(Nicotiana sp.)*	L	14			0%	

Cultivars are listed if they are recorded eaten by wild chimpanzees at ≥1 site, and/or they are harvested in ≥1 range country in areas greater than 1000 ha, according to FAOSTAT (for 2009).

[1] Crops were grouped based on the ‘Indicative Crop Classification’ system which is consistent with the classification used in FAOSTAT [1].

[2] *Musa* spp. includes all edible plantains and sweet bananas.

[3] The part utilised by humans: F = fruit, G = grain, U = underground storage organ (includes tubers, bulbs, rhizomes), P = pith, L = leaf, S = seed, Fl = flower, Fib = fibre, La = latex. While maize is popularly considered a grain, maize kernels are technically fruits.

[4] The number of chimpanzee range countries in which each crop is harvested in an area >1000 ha. Crops that are boxed are ‘important widespread commercial crops’ (present in ≥11 countries).

[5] Percentage ranges indicate the % sites at which ≥1 crop part was confirmed eaten (lower value) and recorded eaten (including unconfirmed records; higher value) by chimpanzees; single values indicate all site records were confirmed. For cashew and tea, chimpanzees do not consume or destroy the part utilised by humans. While we did not calculate the % sites at which chimpanzees eat oil-palm because exclusively wild or naturalised palms are consumed at some sites where chimpanzees have no access to crops (see Methods), FAO data indicate oil-palm is harvested commercially in many range countries. Since oil-palms are an important food for some chimpanzee populations, and may be consumed from cultivated sources, we considered it potentially high conflict. Chimpanzees in northern DRC consume wild coffee berries, but coffee-feeding from cultivated plantations was not reported [2].

[6]

H	HIGH conflict: Important and widespread cash crop (harvested ≥1000 ha in 11 countries or more) and/or staple subsistence crop consumed at ≥25% of chimpanzee sites.
	Potentially HIGH: Non-staple subsistence crop (e.g. domestic fruit) consumed at ≥25% of chimpanzee sites.
L	LOW conflict: Non-staple subsistence crop and/or unimportant cash crop (harvested ≥1000 ha in <11 countries) that is not consumed by chimpanzees.<br>+ indicates an inedible crop (i.e. assumed to be unpalatable raw due to toxic compounds or extreme spiciness); inedible crops were always considered ‘LOW’ conflict.
	Potentially LOW: Important and widespread commercial &/or staple crop not consumed by chimpanzees; or else the part eaten is not utilised by humans and consumption does little damage to the plant.
p	Palatable: Removed from ‘low’ conflict list as the crop is very similar in taste to frequently consumed wild or cultivated food.

Crops that were not classified as high or low conflict according to these definitions were considered ‘intermediate’, accepting that consumption by chimpanzees might create high conflict under certain local conditions (e.g. orange, where it is grown commercially). Note that these conflict classifications are intended as a guide only. See Methods section for discussion of limitations to this approach.

References for Table S1

1. Food and agriculture organization of the United Nations statistical database (FAOSTAT). Available from http://faostat.fao.org/ accessed May 2011.
2. Hicks TC (2010) A chimpanzee mega-culture? Exploring behavioral continuity in *Pan troglodytes schweinfurthii* across northern DR Congo. University of Amsterdam, Netherlands: PhD thesis.
